# Supplementary material for: Non-Alcoholic Fatty Liver Disease, and the Underlying Altered Fatty Acid Metabolism, Reveals Brain Hypoperfusion and Contributes to the Cognitive Decline in APP/PS1 Mice
Source: Metabolites. 2019 May 25;9(5):104. doi: 10.3390/metabo9050104 (PMC6572466; doi:10.3390/metabo9050104)
Supplement: Supplementary file 1 [file metabolites-09-00104-s001.pdf]

Non-Alcoholic Fatty Liver Disease, and the Underlying Altered Fatty Acid Metabolism, Reveals Brain Hypoperfusion and Contributes to the Cognitive Decline in APP/PS1 Mice

Anthony Pinçon, Olivia De Montgolfier, Nilay Akkoyunlu, Caroline Daneault, Philippe Pouliot, Louis Villeneuve, Frédéric Lesage, Bernard I Levy, Nathalie Thorin-Trescaces, Éric Thorin, Matthieu Ruiz.

Online supplemental data

Supplemental table 1: NAFLD altered the lipidome in the brain

List of lipids identified by MS/MS significantly discriminating brain from APP/PS1 mice fed a standard diet (STD) (n=10) or cholesterol-enriched diet (CHOL) (n=10). This list corresponds to lipids selected following untargeted lipidomic analysis using a corrected *P*-value threshold of 0.375 (correspond to an uncorrected *P*-value of 0.015) and a FC >1.2 or <0.83.

Lipid species	Fold change	CHOL *vs.* STD	p value	Corrected p value
hexadecenoyl carnitine	0.78	down	1.1E-02	3.2E-01
Lyso PC 16:1	0.67	down	1.8E-03	1.2E-01
palmitoyl carnitine	0.83	down	8.1E-04	6.9E-02
Lyso PC 16:0	0.55	down	1.5E-02	3.7E-01
Lyso PE 22:5	1.89	up	1.2E-03	9.7E-02
Lyso PC 22:5	1.73	up	2.5E-06	6.6E-04
Lyso PE 22:5	1.60	up	1.2E-06	4.3E-04
FA 22:5	1.58	up	3.0E-05	5.7E-03
PE(16:1_22:6)	0.68	down	7.4E-03	2.5E-01
PS(18:1_22:6)	0.67	down	5.7E-03	2.1E-01
PC(16:1_18:2)	0.61	down	1.4E-04	1.8E-02
PE(16:1_20:4)	0.72	down	2.3E-03	1.4E-01
PC(18:2_20:4)	0.68	down	2.8E-03	1.5E-01
PC(22:5_22:6)	2.55	up	5.7E-09	5.9E-06

PC(18:2_18:2)	0.63	down	6.3E-09	5.9E-06
PE(18:2_20:4)	0.76	down	1.2E-02	3.5E-01
SM(d18:2/18:0)	0.73	down	5.6E-03	2.1E-01
Cer(d18:2/18:0)	0.73	down	1.3E-02	3.7E-01
PE(22:5_22:6)	2.27	up	2.0E-04	2.3E-02
PC(16:0_16:1)	0.78	down	1.4E-03	1.0E-01
PE(16:0_16:1)	0.83	down	3.7E-03	1.7E-01
PE(16:1_18:1)	0.72	down	4.4E-03	1.9E-01
PS(20:1_20:4) or PS(18:0_22:5)	0.70	down	1.4E-02	3.7E-01
PC(16:0_22:5)	3.41	up	1.1E-05	2.6E-03
PC(16:0_24:6)	0.57	down	3.6E-03	1.7E-01
PS(22:5_18:0)	1.50	up	2.8E-03	1.5E-01
DG(16:1_20:4)	0.70	down	5.7E-03	2.1E-01
PC(18:0_22:5)	1.76	up	1.3E-05	2.7E-03
PE(18:0_22:5)	1.49	up	1.9E-03	1.2E-01
DG(16:1_18:1)	0.65	down	4.4E-03	1.9E-01
PC(O-18:0/22:5)	0.47	down	1.6E-02	3.7E-01
CE(22:6)	0.39	down	1.5E-02	3.7E-01
CE(22:6)	0.5	down	4.8E-03	1.9E-01
Lyso PE 22:5	1.62	up	1.5E-04	1.9E-02
PC(18:1_18:2)	0.78	down	4.7E-04	4.7E-02
PS(22:5_18:0)	1.58	up	5.8E-04	5.4E-02
PE(16:0_22:5)	1.61	up	7.3E-04	6.5E-02
PC(19:1_16:0) or PC(17:0/18:1)	1.20	up	1.5E-02	3.7E-01
PE(18:0/22:5)	1.61	up	1.4E-03	1.0E-01

PC: phosphatidylcholine, PE: phosphatidylethanolamine, PS: phosphatidylserine, FA: fatty acid SM: Sphingomyelin, Cer: Ceramide. The symbol “underscore (_)” besides the acyl chain refers to acyl chains for which the “sn” remains to be ascertained although the most likely based on the MS/MS fragmentation profile.

Supplemental table 2: NAFLD reduced the cholesterol precursor desmosterol in the brain

List of cholesterol, its precursors and metabolites identified from the list of MS signals generated by non-targeted lipidomic analysis, and validated using standards, in brain obtained from APP/PS1 mice fed a standard diet (STD) (n=10) or cholesterol-enriched diet (CHOL) (n=10)

Lipid species	Fold change	CHOL *vs*. STD	*p* value	Corrected *p* value
24S-hydroxycholesterol	1.05	up	8.4E-01	9.5E-01
Desmosterol	0.76	down	9.9E-07	4.3E-04
Lathosterol	1.05	up	5.6E-01	8.3E-01
Cholesterol	0.98	down	6.8E-01	8.8E-01

SUPLEMENTAL FIGURES

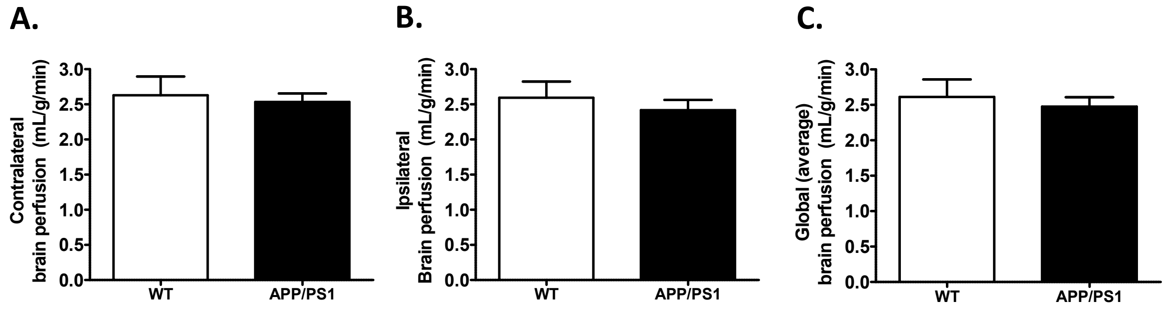

Supplemental Figure 1: Brain perfusion is unchanged in 6-months APP/PS1 mice compared to WT fed with a standard diet

Quantification of (**A**) contralateral, (**B**) ipsilateral and (**C**) global brain perfusion by 7-T IRM in WT and APP/PS1 mice fed a standard diet (STD) diet (n=7 and 10 respectively).

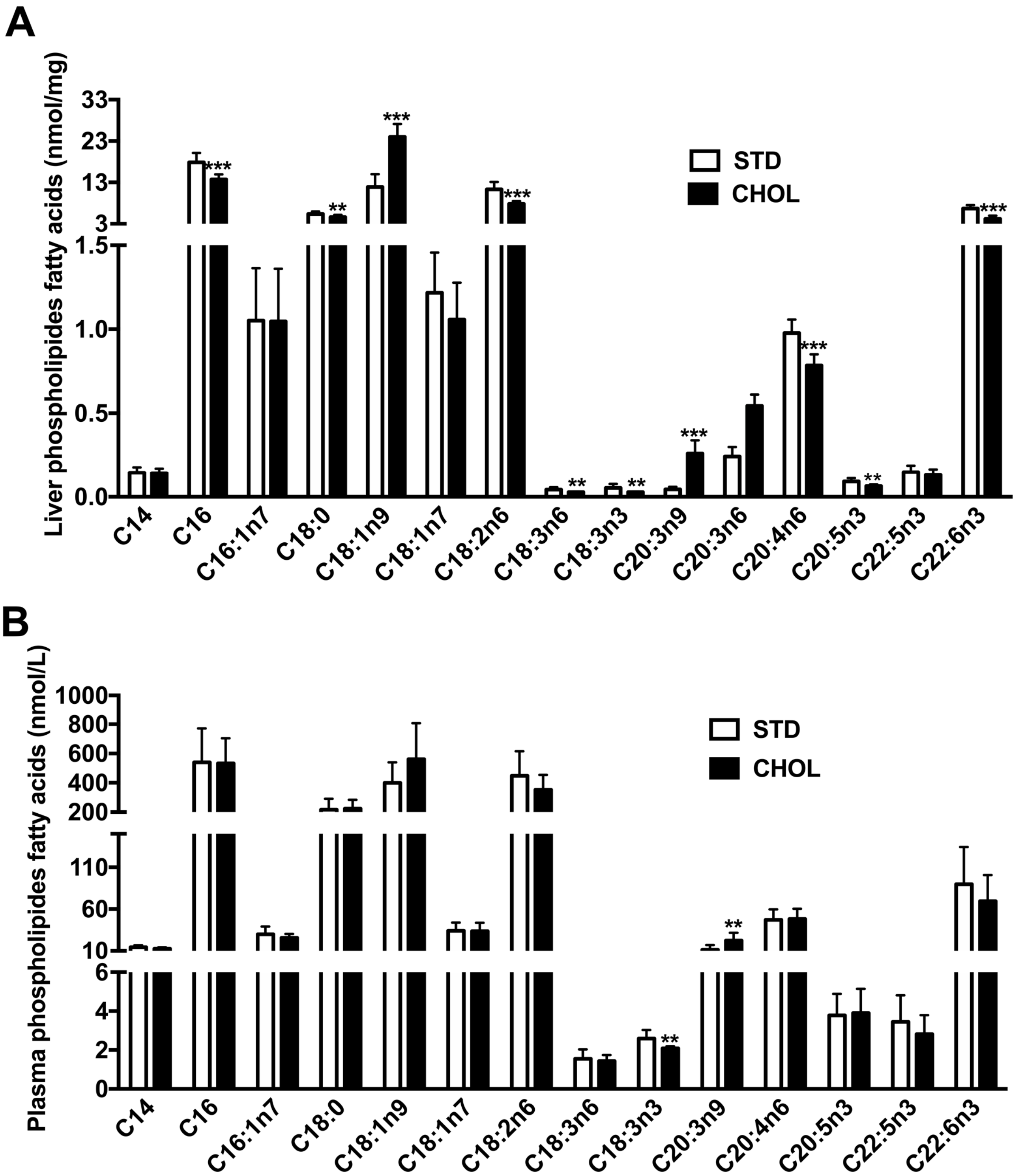

Supplemental Figure 2: NAFLD imbalances fatty acids level in the phospholipids of the liver in APP/PS1 mice. (**A**) Liver and (**B**) plasma fatty acid profiles in phospholipids fractions in

APP/PS1 mice fed a standard diet (STD) or a cholesterol-enriched diet (CHOL). Results are means ± SD. Unpaired t-tests were performed to assess differences between groups; (n=10 animals *per* group). All animals were included in analyses. * $p<0.05$, ** $p<0.01$, *** $p<0.001$.
